# Circular RNA ZNF609 functions as a competitive endogenous RNA to regulate AKT3 expression by sponging miR-150-5p in Hirschsprung's disease

**DOI:** 10.18632/oncotarget.13656

**Published:** 2016-11-26

**Authors:** Lei Peng, Guanglin Chen, Zhongxian Zhu, Ziyang Shen, Chunxia Du, Rujin Zang, Yang Su, Hua Xie, Hongxing Li, Xiaoqun Xu, Yankai Xia, Weibing Tang

**Affiliations:** ^1^ State Key Laboratory of Reproductive Medicine, Institute of Toxicology, School of Public Health, Nanjing Medical University, Nanjing China; ^2^ Key Laboratory of Modern Toxicology, Nanjing Medical University, Ministry of Education, China; ^3^ Department of Pediatric Surgery, Children's Hospital of Nanjing Medical University, Nanjing China

**Keywords:** hirschsprung disease, circular RNA (circRNA), competing endogenous RNAs (ceRNA), proliferation, migration

## Abstract

Research over the past decade suggested critical roles for circular RNAs in the natural growth and disease progression. However, it remains poorly defined whether the circular RNAs participate in Hirschsprung disease (HSCR). Here, we reported that the cir-ZNF609 was down-regulated in HSCR compared with normal bowel tissues. Furthermore, suppression of cir-ZNF609 inhibited the proliferation and migration of cells. We screened out several putative cir-ZNF609 ceRNAs of which the AKT3 transcript was selected. Finally, RNA immunoprecipitation and luciferase reporter assays demonstrated that cir-ZNF609 may act as a sponge for miR-150-5p to modulate the expression of AKT3. In conclusion, these findings illustrated that cir-ZNF609 took part in the onset of HSCR through the crosstalk with AKT3 by competing for shared miR-150-5p.

## INTRODUCTION

Congenital intestinal aganglionosis also known as Hirschsprung disease (HSCR) is a congenital digestive deformity, which resulted from the aberration of enteric neural crest cells (ENCCs) migrating along the intestine [1]. The incidence of congenital intestinal aganglionosis in live births is 1/5000 and the male: female ratio is 4:1 [2]. Up to date, some protein coding genes like RET have been demonstrated to take part in the pathogenesis of HSCR. Recently, evidence have indicated that non-protein coding genes such as miR-218-1 [3], miR-206 [4], miR-192/215 [5] and some other microRNAs (miRNAs), can also result in the occurrence of HSCR by retarding the proliferation and migration of cells. At the same time, some long noncoding RNAs (lncRNAs) are also detected significantly differentially expressed in intestinal tissues with HSCR according to the Microarray analysis [6] which suggests another pathogenic factor for HSCR.

Circular RNA (circRNA) is a novel kind of noncoding RNA that form loop structures without 5′-3′ polarities and polyadenylated tails [7]. Most of the circRNAs are endogenous noncoding RNAs, conserved between different species and showed a higher degree of stability than linear mRNAs [8–14]. CircRNAs take part in the occurrence and process of diseases. For instance, CDR1as has been confirmed to be associated with Parkinson's disease, Alzheimer's disease and tumor evolution and progression [15, 16]. Cir-ITCH has been implicated in esophageal squamous cell carcinoma (ESCC) [17]. On all these counts, circRNAs are proved to be enriched with miRNA binding sites to combine with miRNAs which always be responsible for cellular migration and proliferation [18].

The circular RNA ZNF609 (ID: hsa_circ_0000615 in circBase) locates at chr15:64791491-64792365 and its associated-gene symbol is ZNF609. Cir-ZNF609 expresses sufficiently in the neuron and is indispensable for the growth of central nervous system (CNS) [19]. Lately, some circRNAs have been proposed to function as competing endogenous RNAs to modulate miRNA target gene expression [20]. It remains to be investigated whether cir-ZNF609 can play a role as ceRNA in the incidence of HSCR.

In this research, we hypothesized whether circRNA ZNF609 is expressed abundantly in enteric neuron and is crucial for the development of enteric nervous system (ENS) ENS. Experiments were conducted to unravel the biological roles of cir-ZNF609 and underlying regulation mechanism between cir-ZNF609 and its interactional RNA, which may contribute to the pathogenesis of HSCR.

## RESULT

### Amplification and characteristics of cir-ZNF609

**Figure 1:**
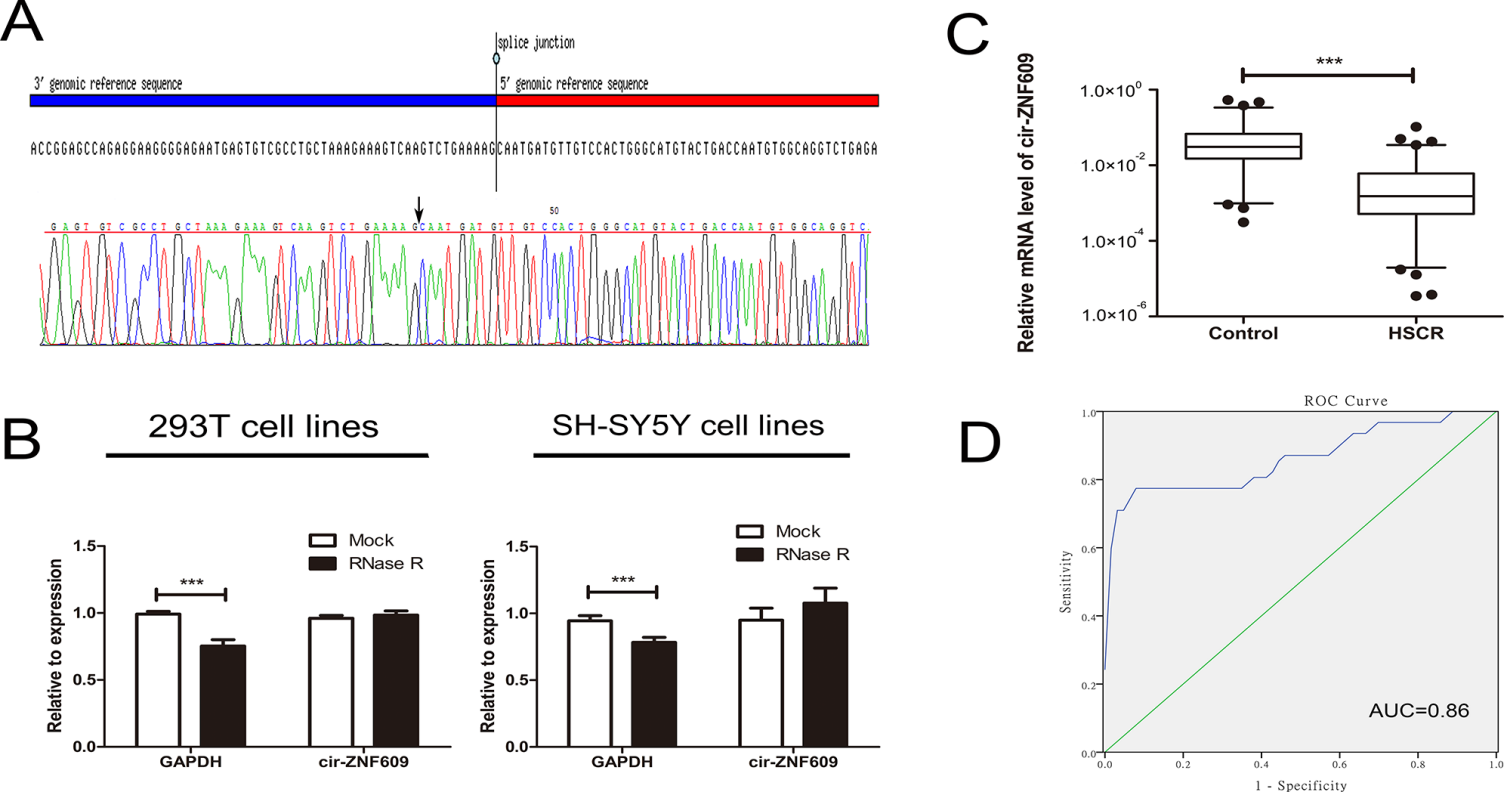
Characteristic and expression of cir-ZNF609 in HSCR (**A**) The sequence of cir-ZNF609 in circBase (upper part) was consistent with the result of Sanger sequencing (lower part). (**B**) The circular RNA was resistant to RNase R treatment in 293T cell lines and SH-SY5Y cell lines. (**C**) The expression of cir-ZNF609 in HSCR tissues (*n* = 80) and control tissues (*n* = 78). Cir-ZNF609 was significantly reduced in patient tissues compared with control tissues. D: Receiver Operating Characteristic (ROC) curve for the cir-ZNF609 to distinguish HSCR cases from controls.

Primers of a circRNA vary from the linear one, for this reason, we analyzed the amplified cir-ZNF609 product to confirm its specificity. Firstly, the melting curve was a single peak (Supplementary Figure 1A), which indicate that there were non-specific amplification. At the same time, the amplified product of cir-ZNF609 was send to sequencing and the result was completely in accordance with the sequence in CircBase (Figure 1A). The outcome showed that cir-ZNF609 existed in intestine and could be amplified by qRT-PCR. To further confirm the characteristics of cir-ZNF609, we used a highly processive 3′ to 5′ exoribonuclease (RNase R enzyme) that does not act on circular RNAs but linear RNAs [21, 22]. As might be expected, the circular RNA was resistant to RNase R treatment in contrast to the linear control RNAs (Figure 1B).

### Expression of cir-ZNF609 in HSCR

The expression of cir-ZNF609 was detected in 80 HSCR samples and 78 control samples by qRT-PCR. The age, gender and body weight of participants was obtained when collecting samples. The ages of HSCR patients and controls were 118.23 ± 9.38 days and 116.70 ± 6.31 days while the body weight was 5.59 ± 0.21 kg and 5.78 ± 0.13 kg separately. There was no significant difference between HSCR cases and normal controls in the clinical information (Table 1).

**Table 1:** Clinical features of study population

Variable	Control (*n*= 78)	HSCR (*n* = 80)	*P*
Age (days,mean,SE)	118.23 (9.38)	116.70 (6.31)	0.88^a^
Weight (kg,mean,SE)	5.59 (0.21)	5.78 (0.13)	0.44^a^
Sex (%)			
Male	56 (71.79)	63 (78.75)	0.31^b^
Female	22 (28.21)	17 (21.25)	

a Student's *t*-test

b Two-sided chi-squared test.

Cir-ZNF609 was shown to be significantly decreased in HSCR, which indicated that cir-ZNF609 might participate in the pathogenesis of HSCR (Figure 1C). Since circRNAs can act as biomarkers of diseases in numerous studies [29, 30], the receiver operating characteristic (ROC) curves analysis was then proceeded to evaluate the diagnostic sensitivity and specificity of the cir-ZNF609 for HSCR. The cutoff value, sensitivity and specificity were 0.0051, 77% and 94%, which were even higher than the sensitivity (70%) and specificity (83%) of the most common method (contrast enema) for the assessment of children with suspected HSCR [23]. The area under the curve (AUC) of cir-ZNF609 was 0.86 exhibited in Figure 1D, which showed that cir-ZNF609 has a potential diagnostic capability.

### Effect of cir-ZNF609 in cell lines

**Figure 2:**
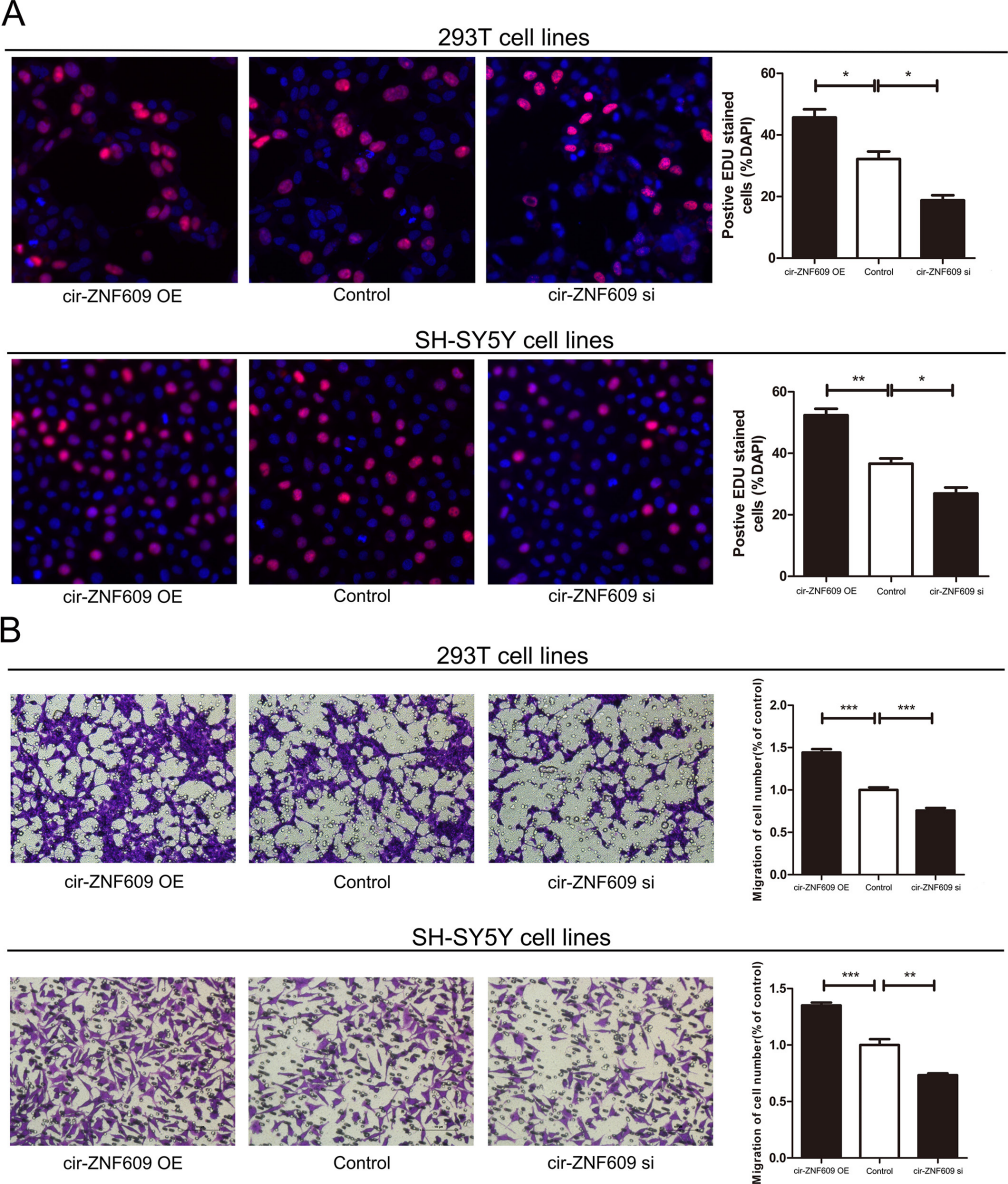
Cytobiology change after treating cells with cir-ZNF609 siRNA (**A**) Human 293T and SH-SY5Y cell lines were transfected with cir-ZNF609 siRNA and over-expression plasmid to regulate its expression levels and cell proliferation was detected using the EDU assay. Knockdown of cir-ZNF609 suppressed cell proliferation and overexpression of cir-ZNF609 have the opposite effect. (**B**) Transwell assay was performed as described in method and indicated that down-regulation of cir-ZNF609 delayed cell migration. However, higher expression of cir-ZNF609 promoted the cell migration. Pictures were captured under a light microscope with the magnification, ×20.

Since cir-ZNF609 was reduced in HSCR tissues, we attempted to suppress its expression to investigate its effects on cells. We then constructed siRNAs targeting cir-ZNF609 and transfected them into 293T cells and SH-SY5Y cells, and found that the expression of cir-ZNF609 could be effectively inhibited by siRNAs (Supplementary Figure 1B) and increased by plasmid (Supplementary Figure 1C). The EDU assays and Transwell assays revealed that the proliferation and migration rate of human 293T cells and SH-SY5Y cells were attenuated dramatically after cir-ZNF609 interference. All these results illustrated that cell migration and cell proliferation rate was sufficiently inhibited by suppression of cir-ZNF609. However, the 293T cells and SH-SY5Y cells transfected with plasmid, which can overexpress the cir-ZNF609 showed an increased number of proliferation and migration cells (Figure 2A and 2B). Flow cytometry demonstrated that the down-regulation of cir-ZNF609 had no effect on cell cycle progression and apoptosis. As shown in Supplementary Figure 1D and 1E.

### Subcellular localization of cir-ZNF609

**Figure 3:**
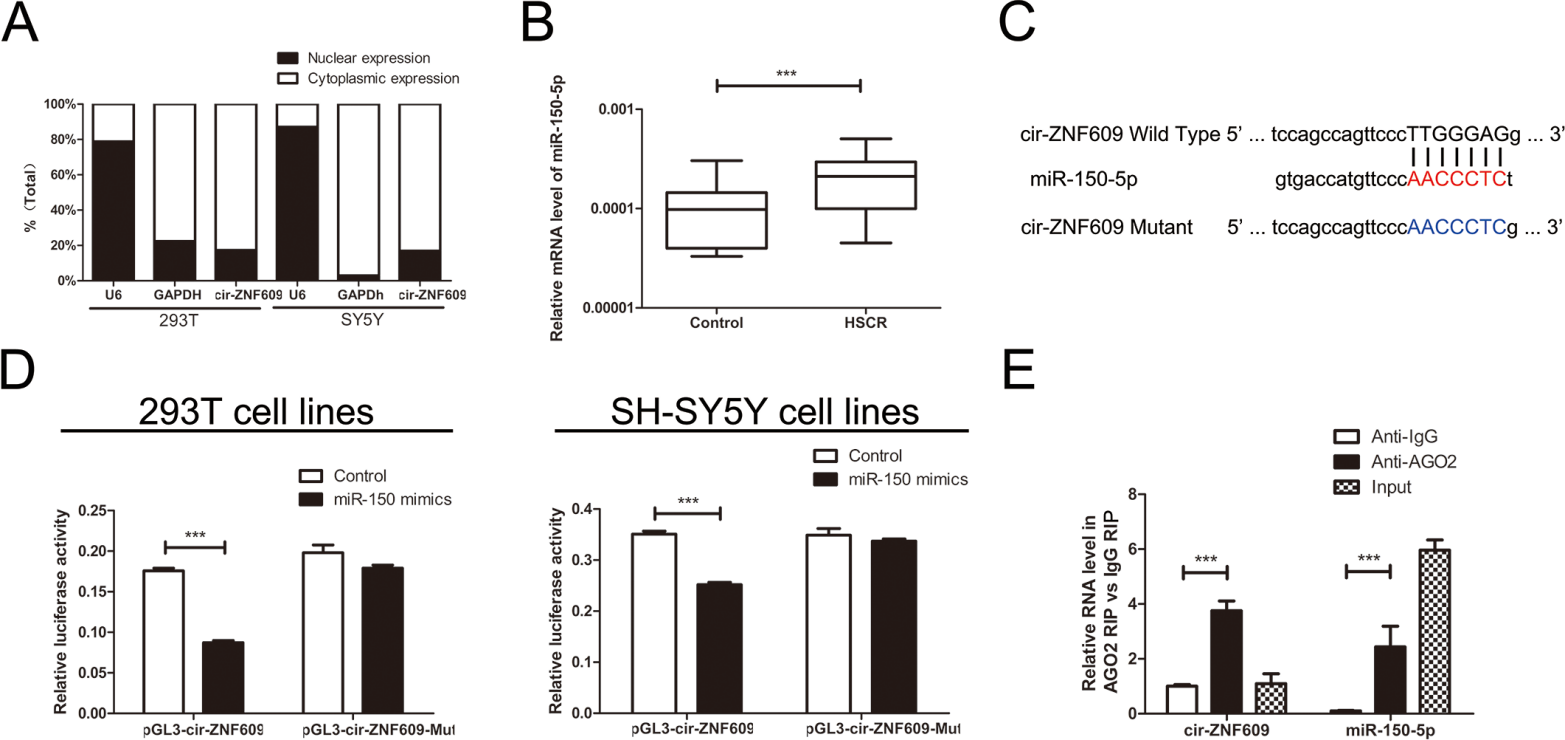
Cir-ZNF609 serves as a sponge for miR-150-5p (**A**) The levels of nuclear control transcript (U6), cytoplasmic control transcript (GAPDH), and cir-ZNF609 were assessed by qRT-PCR in nuclear and cytoplasmic fractions. (**B**) The expression of miR-150-5p in HSCR tissues and control tissues. MiR-150-5p was significantly rose in patient tissues compared with control tissues. (**C**) Sequence alignment of human miR-150-5p with cir-ZNF609. Bottom: mutations in the cir-ZNF609 sequence to create the mutant luciferase reporter constructs. (**D**) Luciferase reporter assay in 293T and SH-SY5Y cells after transfected with negative control or miR-150-5p mimics, renilla luciferase vector pRL-SV40 and the reporter constructs. Both firefly and renilla luciferase activities are measured in the same sample. Firefly luciferase signals were normalized with renilla luciferase signals. (**E**) RNA immunoprecipitation (RIP) experiments were performed using the Ago2 and IgG antibody to immunoprecipitate and primers were used to detect miR-150-5p and cir-ZNF609.

In general, the subcellular localization of noncoding RNAs determines its mode of action. To identify the cellular localization of cir-ZNF609, we separate cells into nuclear and cytoplasmic fractions. The U6 was predominantly found in the nuclear fraction, while the GAPDH level was detected exclusively in the cytoplasmic fraction. Meanwhile, the cir-ZNF609 was detected 82.6% and 83.0% in the cytoplasm fraction in 293T cells and SH-SY5Y cells respectively (Figure 3A). This result revealed that cir-ZNF609 located most in the cytoplasm, which means it could well play a regulative role in the post-transcriptional manner.

### Cir-ZNF609 serves as a sponge for miR-150-5p

Since cir-ZNF609 was down-regulated in HSCR tissues and could suppress cell proliferation and migration. We considered how it participated in the nosogenesis of HSCR. Lately, there has been increasing evidence indicated that circRNAs could function as miRNA sponges [24]. Cir-ZNF609 was predicted to have binding sites with several miRNAs by starBase [25] (http://starbase.sysu.edu.cn/), and miR-150-5p has the highest clipreadNum (Supplementary Figure 1F). The mRNA level of miR-150-5p was detected in HSCR tissues and control tissues, as shown in Figure 3B. To validate the interaction between miR-150-5p and cir-ZNF609, a fragment of cir-ZNF609 including the predicted target site or the mutant target site was conducted to the downstream of the firefly luciferase gene (named as pGL3-cir-ZNF609-Wild and pGL3-cir-ZNF609-Mut) (Figure 3C). The construct was co-transfected with miR-150-5p mimics or negative control into 293T cells and SH-SY5Y cells. Yet, miR-150-5p mimics induced a reduction in relative luciferase expression in pGL3-cir-ZNF609-Wild compared with the negative control. In contrast, there was no difference in the luciferase activity of pGL3-cir-ZNF609-Mut between miR-150-5p mimics and the Control (Figure 3D). These data present that miR-150-5p directly targets cir-ZNF609 *in vitro*. As is well known that miRNA exhibits an important role by binding to Ago2, the core part of the RNA-induced silencing complex (RISC). Therefore, the RNA immunoprecipitation(RIP) was performed on 293T cell by using antibodies against Ago2 to evaluate whether cir-ZNF609 correlates with RISC. qRT - PCR was conducted to measure RNA levels in immunoprecipitates. Cir-ZNF609 was enriched in Ago2-containing immunoprecipitates compared with control immunoglobulin G (IgG) immunoprecipitates. Equally, the miRNA-150-5p level of Ago2-containing immunoprecipitates was detected higher than that of control IgG immunoprecipitates (Figure 3E).

### Cir-ZNF609 regulates the miR-150-5p target, AKT3

**Figure 4:**
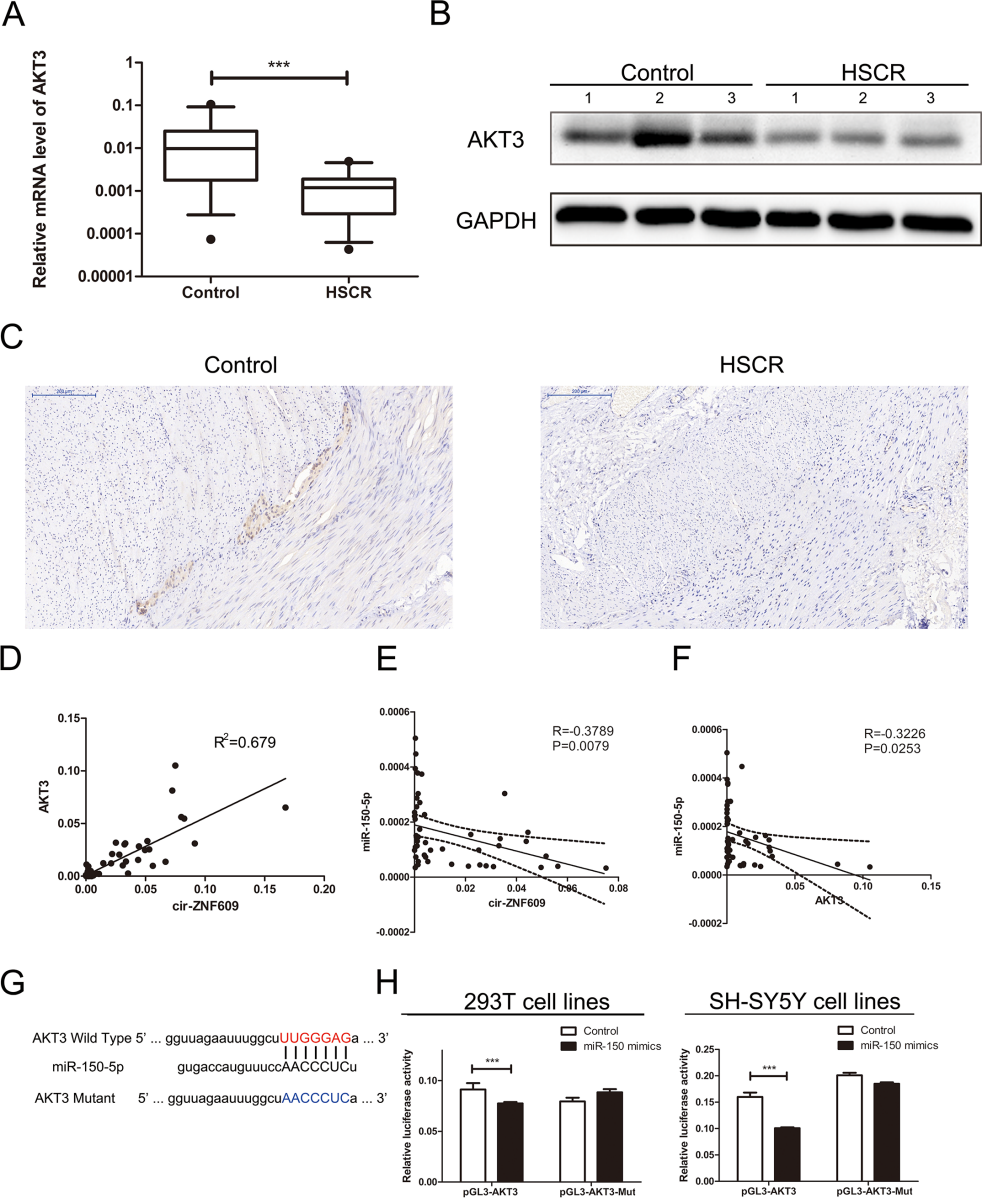
Cir-ZNF609 regulates the miR-150-5p target, AKT3 (**A**) Relative expression of AKT3 in HSCR tissues in comparison with control tissues. AKT3 was significantly reduced in patient tissues. (**B**) Protein level of AKT3 in HSCR tissues and normal control samples was detected by Western Blot. (**C**) Immunostaining of AKT3 protein in stenotic segment and control tissues. Immunostaining of AKT3 was negative in HSCR tissues and was positive in control tissues. (**D**) Bivariate correlation analysis of the relationship between cir-ZNF609 and AKT3 expression level. (**E**) There was a significant negative correlation between the expression level of cir-ZNF609 and the expression level of miR-150-5p in the same paired intestinal samples (*P* = 0.0079, Pearson). (**F**) There was a significant negative correlation between the expression level of AKT3 and the expression level of miR-150-5p in the same paired intestinal samples (*P* = 0.0253, Pearson). (**G**) The putative miRNA binding sites in the AKT3 sequence. The putative miRNAs recognition sites was cloned downstream of the luciferase gene and named pGL3-AKT3-Wild. Bottom: mutations in the AKT3 sequence to create the mutant luciferase reporter constructs named pGL3-AKT3-Mut. (**H**) Left: The luciferase reporter in 293T cell lines. Right: the luciferase reporter in SH-SY5Y cells. Luciferase activity was determined using the dual luciferase assay and shown as the relative luciferase activity normalized to renilla activity.

Among the putative target genes of miR-150-5p from bioinformatic prediction (DIANA, miRanda, PicTar, PITA), three genes were selected after functional analysis, namely *AKT3* (AKT serine/threonine kinase 3), *FOXO4* (forkhead box O4) and *ELK1* (ETS transcription factor). AKT3 was associated with glioblastoma; ELK1 participated in neurotrophin TRK receptor signaling pathway and FOXO4 can negatively regulate cell proliferation. Then the mRNA level of predicted mRNAs was detected in HSCR samples and controls. AKT3 was down-regulated in HSCR patients compared with controls (Figure 4A). However, there were no significant differences in the mRNA levels of FOXO4 and ELK1 between HSCR tissues and controls (Supplementary Figure 1G). The protein level of AKT3 was in parallel with the mRNA levels (Figure 4B), and so was the IHC (Figure 4C). We processed Bivariate correlation analysis to evaluate the expression of cir-ZNF609, miR-150-5p and AKT3 in colon tissues containing both normal and pathological biological tissues (Figure 4D, 4E, 4F). The results showed that AKT3 positively correlated with levels of cir-ZNF609 and miR-150-5p expression was inversely correlated with cir-ZNF609 and AKT3 expression in control and HSCR tissues. To further study whether miR-150-5p interacts with AKT3, the 293T cells and SH-SY5Y cells were transfected with plasmid pGL3-AKT3-Wild and pGL3-AKT3-Mut, which contain the predicted binding site and mutant binding site (Figure 4G). The luciferase activity was inhibited by the miR-150-5p mimics in cells transfected with the pGL3-AKT3-Wild. Meanwhile, the luciferase activity showed no obvious changes in cells transfected with pGL3-AKT3-Mut combined with miR-150-5p mimics or Control (Figure 4H). In general, the findings proved that AKT3 is a direct target of miR-150-5p.

**Figure 5:**
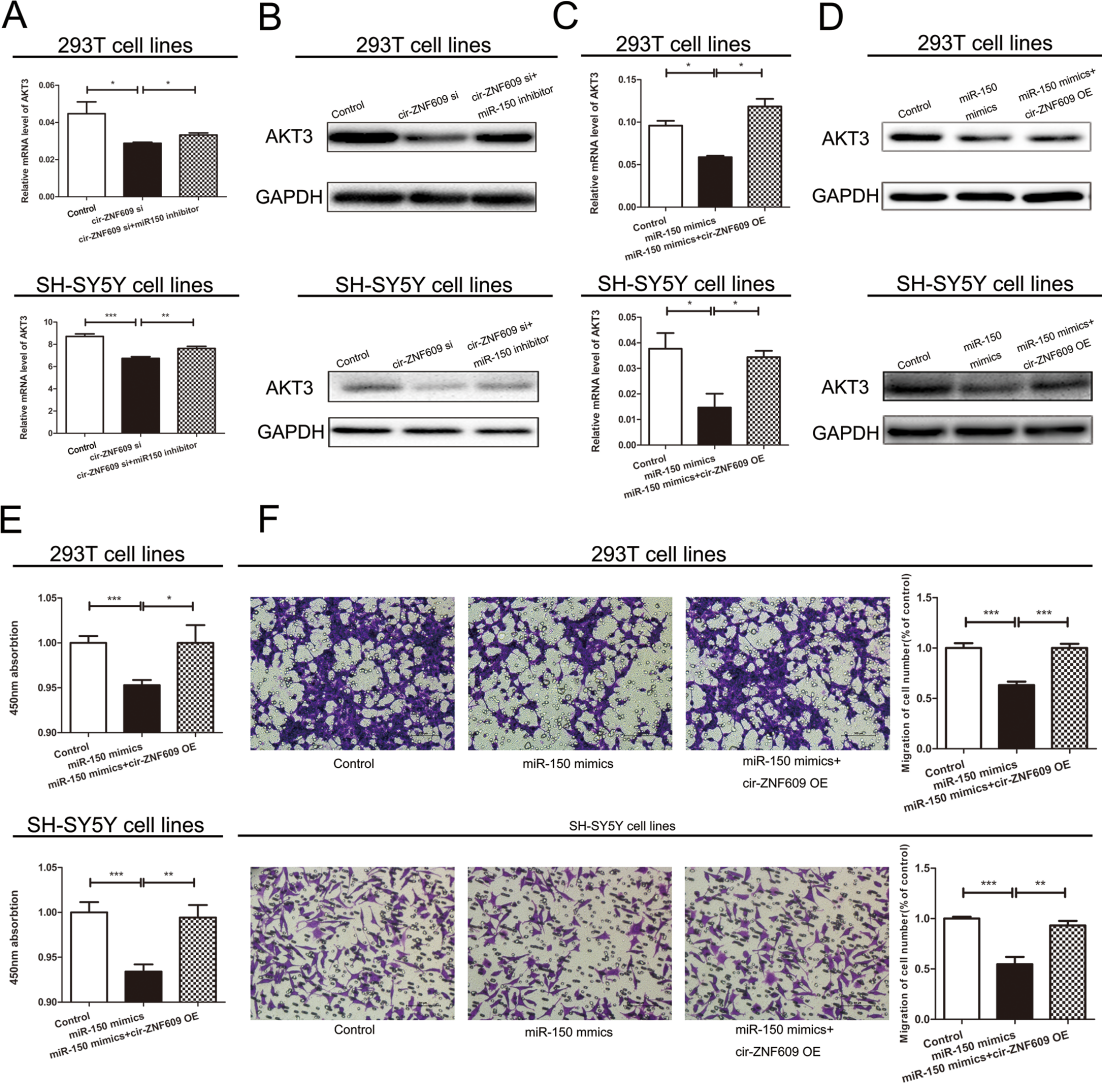
Cir-ZNF609-miR-150-5p regulatory loop is critical for cell function (**A**) cir-ZNF609 siRNA with or without miR-150-5p inhibitor was transfected into 293T cells and the mRNA level of AKT3 was evaluated by qRT-PCR. (**B**) Western blot analysis of AKT3 protein level following treatment of 293T and SH-SY5Y cells with cir-ZNF609 siRNA or miR-150-5p inhibitor. GAPDH was used as control. (**C**) Two types of cells were transfected with miR-150-5p with or without cir-ZNF609 overexpress plasmid and qRT-PCR was used to detect the relative mRNA levels of AKT3 compared with controls. (**D**) Relative protein level of AKT3 when transfected with miR-150-5p mimics and reversed by cir-ZNF609 expression plasmid. (**E**and **F**) CCK8 assay and Transwell assays were performed to determine the proliferation and migration of miR-150-5p transfected cells and treated with miR-150-5p mimics plus cir-ZNF609 expression plasmid.

To determine whether cir-ZNF09 could regulate the AKT3 expression by combining with miR-150-5p, we analyzed the mRNA and protein levels of AKT3 in cells of which the expression of cir-ZNF609 and miR-150-5p were contrasted to normal. As is shown in Figure 5A and 5B, the silence of cir-ZNF609 resulted in a reduction of AKT3 in both mRNA and protein levels. Meanwhile, the expression of AKT3 was elevated in cells treated with miR-150-5p inhibitor. Compared with the control cells, the expression of AKT3 was inhibited by miR-150-5p mimics and can be reversed by cir-ZNF609 plasmid in both mRNA and protein level (Figure 5C and 5D). Collectively, these results uphold that cir-ZNF609 regulates AKT3 by harboring a target site for the same miRNA, miR-150-5p.

### Cir-ZNF609-miR-150-5p regulatory loop is critical for cell function

We next checked whether miR-150-5p was participated in cell proliferation and migration. The level of miRNA was increased by miRNA mimics and decreased by miRNA inhibitor, the efficiency of miR-150-5p mimics and inhibitor was evaluated by qRT-PCR (Supplementary Figure 1H). The migration and proliferation ability of cells deal with miR-150-5p mimics were significantly suppressed when compared with cells deal with Control. However, cir-ZNF609 overexpression could partially abrogate miR-150-5p repression effect (Figure 5E and 5F). Above all, these results reflected the interplay among cir-ZNF609, miR-150-5p, and AKT3.

## DISCUSSION

Circular RNAs (circRNAs), which form covalently closed continuous loops, often have complex disease-, tissue- and stage-specific expression patterns, indicating particular functions of circRNAs in diseases and development [26, 27]. A recent paper revealed the abundance and function of circRNAs in the central nervous system, including the cir-ZNF609 [19]. The enteric nervous system (ENS) is the largest element of the autonomic nervous system and is called the ‘second brain’ [28]. The dysplasia of ENS is responsible for many disorders including HSCR and there are few researches about the circRNAs in ENS and HSCR.

In this study, the expression of cir-ZNF609 was detected in HSCR colon tissues and the function of cir-ZNF609 in 293T cells and SH-SY5Y cells was identified by gain- and loss-of-function methods. These results showed that cir-ZNF609 was down-regulated in HSCR stenotic segment compared with gut from patients without HSCR or other enteric neural malformations. Furthermore, it is shown that cir-ZNF609 had a higher degree of stability than linear RNAs through the RNase R digestion experiment. Moreover, the migration and proliferation of cells treated with siRNA against cir-ZNF609 were inhibited. Besides, the cir-ZNF609 plasmid has the opposite functions. All these findings indicated that perturbation of cir-ZNf609 may contribute to the HSCR progression and revealed a new possible etiology of HSCR.

Recently, in hepatocellular carcinoma [29] and gastric cancer [30], researchers found that circRNAs could be a novel type of biomarker. Therefore, cir-ZNF609 may function as a biomarker for its expression and particularly the stability, the sensitivity and specificity were higher than those for CE (70% and 83%). But it is more significant to diagnose HSCR by cir-ZNF609 in serum than using the tissue samples. A part of the patient received surgery when diagnosed, but the prognosis was always undesirable [31]. So we are likely to pay attention to use the cir-ZNF609 to predict the prognosis and endeavor to improve the prognosis.

CircRNAs took part in human diseases through varieties of mechanisms. We detected the subcellular localization of cir-ZNF609 in cells and found the cir-ZNF609 located in the cytoplasm mostly, which promotes the possibility that it works at the post-transcriptional level. Several groups showed that circRNAs serve as a miRNA sponge in Cartilage Degradation [32], heart failure [33], diabetes [34] and so on. We hypothesized that cir-ZNF609 might sequester miRNAs thereby preventing their target RNAs from repression. To support this view, we predicted the miRNA by bioinformatics analysis and employed dual-luciferase reporter gene assay to validate the miRNA combined with cir-ZNF609. As was expected, miR-150-5p could diminish the fluorescence of pGL3-cir-ZNF609-Wild and the Ago2 antibody could pull down miR-150-5p with cir-ZNF609. These results demonstrated a combination between the miRNA and circRNA. Moreover, higher expression of miR-150-5p by mimics inhibited the cell migration and proliferation, which was just consistent with the representation of knockdown of cir-ZNF609 expression. According to previous research, in hepatocellular carcinoma, miR-150 suppresses cell proliferation and metastasis by inhibiting the GAB1-ERK axis [35]. Taken together, these results made the guess a certainty that cir-ZNF609 influenced cell functions by binding to miR-150-5p.

Then we took miR-150-5p for further study, with a particular focus on AKT3, which was predicted as the possible target gene. The protein encoded by AKT3 is a member of the AKT and is involved in variety of biological processes including cell proliferation, differentiation, apoptosis, tumorigenesis. In glioblastoma, it promotes the progression [36] and knockdown of AKT3 suppresses the T98G cells invasiveness [37]. Targeting AKT3 may be an effective approach to inhibit the growth of triple-negative breast cancer [38]. In our study, AKT3 is confirmed to be a validated target gene of miR-150-5p by luciferase assay. Moreover, qRT-PCR, WB and IHC revealed that AKT3 was down-regulated in HSCR. Whether there is a specific crosstalk between cir-ZNF609 and AKT3 as well as miR-150-5p remains uncertain. Consistent with cir-ZNF609 sequestration of miR-150-5p, we found that the suppression of cir-ZNF609 reduced the expression of AKT3, while lower expression of miR-150-5p restored AKT3 protein synthesis. All these findings endorse our speculation that cir-ZNF609 gives an impetus to cellular proliferation and migration by controlling miRNA activity, which attenuates the expression of AKT3.

In conclusion, the current study showed that there is an aberrant expression of cir-ZNF609 in HSCR intestine tissue, and lower cir-ZNF609 suppresses cell migration and proliferation in 293T cell lines and SH-SY5Y cell lines. Generally, all our results support the hypothesis that cir-ZNF609 acts as a miRNA sponge and positively affect the expression of the miRNA target gene AKT3. Also, it provides us a new way to diagnose the HSCR.

## MATERIALS AND METHODS

### Tissue collection

This research was approved by the Institutional Ethics Committee of Nanjing Medical University. All the samples were collected from patients signed a consent form. All the HSCR colon tissues were gathered from Nanjing Children's Hospital between 2011 and 2016 and then stored at −80°C. The HSCR patients were eventually diagnosed via postoperative pathological analysis of excision. Negative controls were from patients affirmed to be without HSCR or other enteric neural malformations. Patients' clinical information are summarized in Table 1.

### RNA isolation and qRT-PCR

Total RNA was extracted from each tissue and cell line according to the manufacturer's instructions by Trizol reagent (Life Technologies, CA, US). The RNA quantity control and concentration detection were achieved using NanoDrop 2000 Spectrophotometer (Thermo Scientific, Wilmington, DE, USA). We used Reverse Transcription Kit (Takara, Tokyo, Japan) to reverse transcription the total RNAs (500 ng) and then employed qRT-PCR on the ABI 7900HT (Applied Biosystems) to measure the expression of circRNA, mRNA and miRNA. The PCR reaction conditions were 95°C for 30s, then 40 cycles of 95°C for 5s and 60°C for 30s. Finally, annealing and extension at 95°C for 15s, 60°C for 60s and 95°C for 15s. All experiments were repeated three times. Fold changes in expression were calculated using 2^−ΔCt^. All primers used in this study are listed in Table 2.

**Table 2:** Sequences of primers for qRT-PCR and siRNA related sequence

Name		Sequence
Cir-ZNF609	Forward	5′- CAGCGCTCAATCCTTTGGGA-3′
	Reverse	5′- GACCTGCCACATTGGTCAGTA-3′
AKT3	Forward	5′- TGAAGTGGCACACACTCTAACT-3′
	Reverse	5′- CCGCTCTCTCGACAAATGGA-3′
FOXO4	Forward	5′- CTTTCTGAAGACTGGCAGGAATGTG-3′
	Reverse	5′- GATCTAGGTCTATGATCGCGGCAG-3′
ELK1	Forward	5′- GGCTACGCAAGAACAAGACCA-3′
	Reverse	5′- CCTCAGGGTAGGACACAAACT-3′
GAPDH	Forward	5′-GCACCGTCAAGGCTGAGAAC-3′
	Reverse	5′-GGATCTCGCTCCTGGAAGATG-3′
U6	Forward	5′-CTCGCTTCGGCAGCACA-3′
	Reverse	5′-AACGCTTCACGAATTTGCGT-3′
miR-150-5p	Forward	5′-ACACTCCAGCTGGGTCTCCCAACCCTTGTA-3′
	Reverse	5′-CTCAACTGGTGTCGTGGAGTCGGCAATTCAGTTGAG CACTGGTA-3′
Cir-ZNF609 siRNA	sense	5′-GUCAAGUCUGAAAAGCAAUGATT-3′
	antisense	5′-UCAUUGCUUUUCAGACUUGACTT-3′
miR-150 mimics	sense	5′-UCUCCCAACCCUUGUACCAGUG-3′

### RNase R digestion

Total RNA (5 ug) was incubated 15 min at 37°C with 3U/ug of RNase R (Epicentre Biotechnologies). The RNase R digestion reaction was performed twice following previously published procedures.

### Sanger sequencing

The amplification products were inserted into a T-vector for Sanger sequencing to determine their full-length. The divergent primers were designed to confirm the back-splice junction of cir-ZNF609: 5′-CAGCGCTCAATCCTTTGGGA-3′ (sense) and 5′-GACCTGCCACATTGGTCAGTA-3′ (antisense). The primers were synthesized by Invitrogen (Shanghai, China), and Sanger sequencing was performed by Realgene (Nanjing, China).

### Protein extraction and Western blotting

RIPA buffer containing protease inhibitors were used to extract total proteins from tissues and cultured cells and the concentrations were measured by bicinchoninic acid (BCA) solution (Beyotime, Nantong, China). The Western Blot was performed under the standard program and the primary antibodies, anti-GAPDH, as well as the secondary antibodies, including anti-rabbit HRP-linked and anti-mouse HRP-linked were from Beyotime (Nantong, China). Besides, anti-AKT3 was from lifesbiology(Wuxi, China) .

### Cell culture and transfection

Human 293T cell and SH-SY5Y cell were obtained from American Type Culture Collection (ATCC, Manassas VA, USA) and were cultured in DMEM (Hyclone, UT, USA), mixed with 10% fetal bovine serum (10% FBS), penicillin (100 U/ml), and streptomycin (100 μg/mL) at 37°C, 5% CO2. The siRNA against cir-ZNF609, miR-150-5p mimics and negative controls (Table 2) were purchased from GenePharma(Shanghai, China). All of the transfection experiments following the manufacturer's instructions need Lipofectamine 2000 Reagent (Invitrogen, CA, USA).

### Dual-luciferase reporter assay

The binding site of cir-ZNF609 and AKT3 called cir-ZNF609-Wild, cir-ZNF609-Mut and AKT3-Wild, AKT3-Mut were inserted into the KpnI and SacI sites of pGL3 promoter vector (Realgene, Nanjing,China) in Dual-luciferase reporter assay. Firstly, Cells were plated on 24-well plates. Then 80 ng plasmid, 5 ng renilla luciferase vector pRL-SV40, 50 nM miR-150-5p mimics and negative control was transfected into cells by applying lipofectamine 2000 (Invitrogen, Shanghai, China). Cells were collected and measured following the manufacturer's instructions by using the Dual-Luciferase Assay (Promega, Madison, WI, USA) after 48 h transfection. All experiments were repeated three times independently.

### Cell migration

Cells were cultured on 6-well plates and transfected with the cir-ZNF609 siRNAs, miR-150-5p mimics and negative control. About 100 μL cell suspension with serum-free medium was seeded in the upper chamber, and 600 μL medium with 10% fetal bovine serum was seeded in the lower chamber. After transfected 36 h, cells were dyed with crystal violet staining solution (Beyotime, Nantong, China), counted and photographed under 40× magnification (five views per well). All experiments were repeated three times independently.

### Cell proliferation

Cells were plated on 96-well plates and after incubation 1h with CCK8(Beyotime, Nantong, China), the TECAN infinite M200 Multimode microplate reader (Tecan, Mechelen, Belgium) was used to measure the absorbance at 450 nm. Also, the EDU assay was taken to detect the proliferation of cells. All experiments were repeated three times independently

### RNA Binding protein immunoprecipitation (RIP) assay

RNA immunoprecipitation (RIP) experiments were performed by the Magna RIP Kit (Millipore, Bedford, MA) according to the manufacturer's instructions. The AGO2 antibody used for RIP was clone ab32381 (Abcam, Shanghai, China).

### Statistical analysis

All experiments were performed in triplicate. Chi-square tests and Student's *t*-test were used to evaluate statistical differences in demographic and clinical characteristics. Receiver operating characteristic (ROC) curves analysis was performed to investigate the association between HSCR and cir-ZNF609 expression levels. Results were considered statistically significant at *P* < 0.05.

## SUPPLEMENTARY MATERIALS


