# Correction: Circular RNA ZNF609 functions as a competitive endogenous RNA to regulate AKT3 expression by sponging miR-150-5p in Hirschsprung’s disease

**DOI:** 10.18632/oncotarget.26963

**Published:** 2019-05-14

**Authors:** Lei Peng, Guanglin Chen, Zhongxian Zhu, Ziyang Shen, Chunxia Du, Rujin Zang, Yang Su, Hua Xie, Hongxing Li, Xiaoqun Xu, Yankai Xia, Weibing Tang

**Affiliations:** ^1^ State Key Laboratory of Reproductive Medicine, Institute of Toxicology, School of Public Health, Nanjing Medical University, Nanjing China; ^2^ Key Laboratory of Modern Toxicology, Nanjing Medical University, Ministry of Education, China; ^3^ Department of Pediatric Surgery, Children’s Hospital of Nanjing Medical University, Nanjing China; ^*^ These authors contributed equally to this work

**This article has been corrected:** Due to an error during image assembly, in figure 2B the images shown for the 293T cell lines are incorrect. The proper images are shown below. The authors declare that these corrections do not change the results or conclusions of this paper.

Original article: Oncotarget. 2017; 8:808–818 . https://doi.org/10.18632/oncotarget.13656

**Figure 2:**
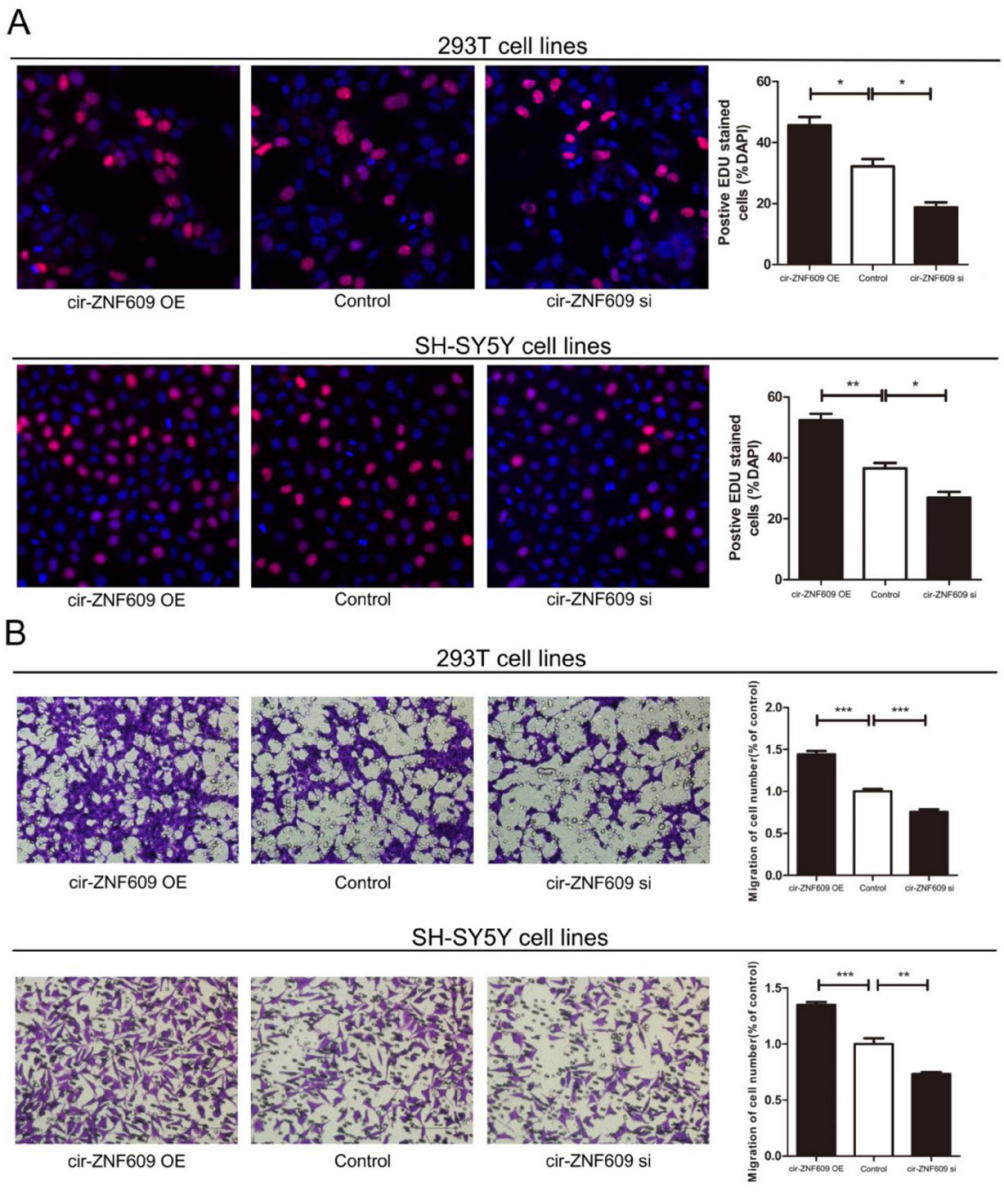
Cytobiology change after treating cells with cir-ZNF609 siRNA. (**A**) Human 293T and SH-SY5Y cell lines were transfected with cir-ZNF609 siRNA and over-expression plasmid to regulate its expression levels and cell proliferation was detected using the EDU assay. Knockdown of cir-ZNF609 suppressed cell proliferation and overexpression of cir-ZNF609 have the opposite effect. (**B**) Transwell assay was performed as described in method and indicated that down-regulation of cir-ZNF609 delayed cell migration. However, higher expression of cir-ZNF609 promoted the cell migration. Pictures were captured under a light microscope with the magnification, ×20.

