# Supplementary material for: Circular RNA ZNF609 functions as a competitive endogenous RNA to regulate AKT3 expression by sponging miR-150-5p in Hirschsprung's disease
Source: Oncotarget. 2016 Nov 26;8(1):808–18. doi: 10.18632/oncotarget.13656 (PMC5352198; doi:10.18632/oncotarget.13656)
Supplement: Supplementary file 1 [file oncotarget-08-808-s001.pdf]

Circular RNA ZNF609 functions as a competitive endogenous RNA to regulate AKT3 expression by sponging miR-150-5p in Hirschsprung's disease

Supplementary Materials

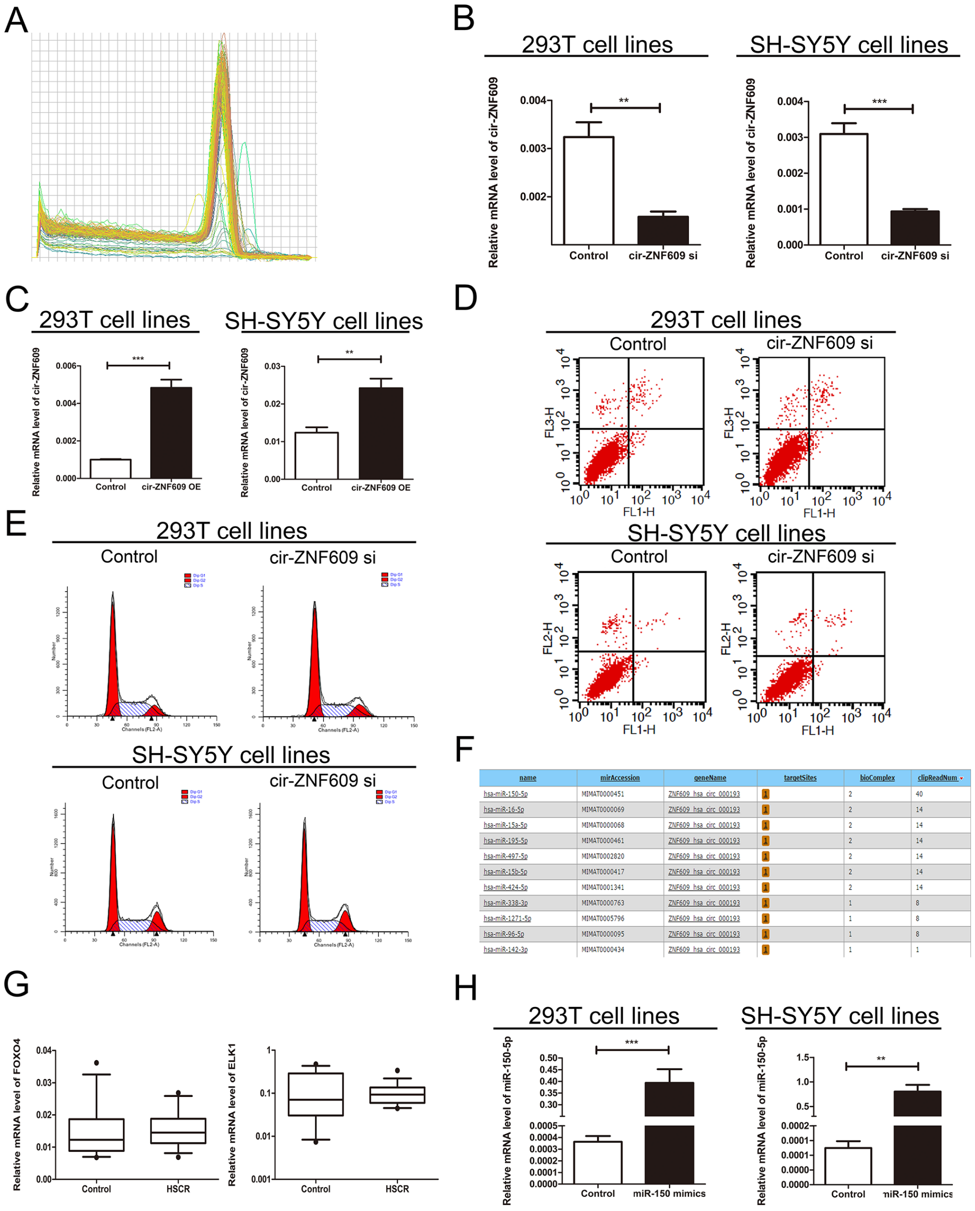

Supplementary Figure S1: (**A**) The melting curve of cir-ZNF609 amplified by qRT-PCR. (**B**) qRT-PCR was used to test the effect of cir-ZNF609 siRNA in 293T cells and SH-SY5Y cells. (**C**) The mRNA level of cir-ZNF609 was detected by qRT-PCR after treat with cir-ZNF609 over-expressing vector. (**D**) Apoptosis rate induced by cir-ZNF609 siRNA in two cell lines was detected by FACS. There was no significant difference between cells treated with siRNA and negative control. (**E**) Cell cycle was detected by BD Biosciences FACS Calibur Flow Cytometry after treated with cir-ZNF609 siRNA which didn't cause cell cycle arrest. (**F**) The forecasting miRNAs interact with cir-ZNF609 were seeked in starBase. (**G**) The expression levels of FOXO4 and ELK1 were detected in HSCR tissues and control tissues. (**H**) Two types of cells were transfected with miR-150-5p mimics and qRT-PCR was used to detect the miRNA levels compared with controls.
